# Economic evaluation of passive monitoring technology for seniors

**DOI:** 10.1007/s40520-019-01323-2

**Published:** 2019-09-14

**Authors:** John E. Schneider, Jacie Cooper, Cara Scheibling, Anjani Parikh

**Affiliations:** 1 Avalon Health Economics, 26 Washington Street, 3rd Floor, Morristown, NJ 07960 USA; 2 Boston Strategic Partners, Boston, MA USA

**Keywords:** Older adults, Health-related quality of life, Passive monitoring technology, Nursing homes, Economic value, Cost–benefit

## Abstract

**Background:**

Advances such as passive monitoring technology (PMT), which provides holistic supervision of chronically ill and elderly patients, enable and support improved monitoring and observation, thus empowering the growing population of older adults to live more independently while lowering health care expenses.

**Aims:**

This study develops a conceptual model to estimate the potential savings associated with PMT.

**Methods:**

We first develop a conceptual model to identify the main cost variables associated with independent living, focusing on three pathways: (1) PMT, (2) independent living supported by the current standard of care, and (3) facility-based care. We examined the impact on three outcomes [i.e., health care costs, institutional costs, and health-related quality of life (HRQoL)] along each of the three care pathways (i.e., PMT, independent living supported by the standard of care, and facility-based care) and developed a cost-benefit model to calculate the net costs and benefits associated with each care pathway.

**Results:**

The cost–benefit model showed savings between approximately $425 per-member per-month (PMPM) for those using PMT compared to those on the standard of care pathway. Sensitivity analysis demonstrated that a 5% increase in nursing home utilization generates cost savings of more than 30% PMPM.

**Discussion:**

The total projected cost savings for individuals on the PMT arm are projected to be more than $425 PMPM, with annual savings of $5069 per-person per-year, and over $5.1 million for a target population of 1000 individuals.

**Conclusions:**

The cost calculations in our cost–benefit simulation model clearly demonstrate the value of PMT and show the potential value to payers and integrated delivery systems in offering PMT to individuals who are likely to benefit the most from the services.

**Electronic supplementary material:**

The online version of this article (10.1007/s40520-019-01323-2) contains supplementary material, which is available to authorized users.

## Introduction

By 2050, the global population of persons over the age of 60 is expected to be between 1.5 and 2 billion [1, 2]. Moreover, the proportion of individuals over the age of 80 is simultaneously and disproportionately rising. The United Nations estimates that over 400 million people will have reached 80 years of age by 2050 [3]. Changes in societal trends have also led to smaller, non-traditional families, creating a decline in social support. The consequence of demographic aging is the existence of many single-dwelling elderly people in our communities [4].

Advances in health technology, such as communication and monitoring systems, have enabled older adults to lead independent lifestyles [5]. Technology-integrated homes and passive monitoring technology (PMT) provide complete supervision of chronically ill and elderly patients using information and communication technology tools in a comfortable environment. These technologies enable and support improved monitoring and observation, which in turn improve the efficiency and timing of patient access to the healthcare delivery system, and enable seniors to live more independently with a sense of security and reduce the feelings of loneliness and isolation as society advances [6].

There are several examples of PMT; in this research, we focus on one that GreatCall has developed referred to as Lively^®^ Home and Lively^®^ Mobile (formerly Healthsense; both referred to as “LHM”) products to help prevent major health events for seniors living alone. The LHM algorithm allows passive monitoring of subscribers’ activities of daily living (ADLs) and generates actionable alerts using data from sensors. GreatCall nurses triage the alerts and validate the data, which are then transmitted to the health plan care manager, who assesses the generated data and contacts the senior. This passive monitoring activity allows seniors to comfortably address the issue causing a change in their daily activity.

## Background

One consequence of longer life expectancy is the increase in the rise in the number of older adults living with chronic morbidities and disabilities [1]. Approximately 75% of preventable health costs are attributable to chronic disease, a large percentage of which can be avoided through earlier diagnosis followed by monitoring and surveillance [7]. Throughout the process of aging, multiple age-dependent diseases can occur, such as dementia, stroke, vascular complications of atherosclerosis, osteoarthritis, cancers, incontinence, osteoporosis, falls, fractures, and bedsores [8]. More than 50% of the older adult population suffers from at least two concurrent chronic diseases and functional decline resulting in decreased efficiency, disability, and poor quality of life which is an increasingly burdensome problem for older adults [9, 10].

An important and increasingly recognized pathology in the elderly is “frailty syndrome.” The development of frailty involves declines in energy production and utilization as well as declines in the body’s “repair systems,” thereby resulting in impaired function of a number of physical and physiological systems [11]. Frailty in older adults also increases the patients’ likelihood of experiencing major adverse health outcomes, such as acute diseases, falls, disability, emergency room (ER) utilization, inpatient hospitalization, institutionalization, and mortality. A study of 5000 older adults, for example, found that the diagnostic criteria for frailty syndrome were met by 7% of people over 65 years of age and 30% of those aged 80 years or older [12].

There are relatively few randomized controlled trials (RCTs) or economic analysis available to support the clinical or cost-effectiveness of current models of PMT; however, there is a body of research on telemedicine and telehealth, both of which constitute what were essentially “first-generation” PMT. This research generally shows that such approaches are beneficial to older adults, especially those with chronic conditions such as cardiovascular diseases, diabetes, cancer, chronic obstructive pulmonary disease (COPD), cognitive decline, and decline in motor skills resulting in falls and other avoidable accidents. [2, 13–17]. Some recently published scientific publications indicate that there are economic benefits of use of telecare solutions [18–21]. For example, a six-month home telemonitoring program for patients with chronic obstructive pulmonary disease (COPD) showed a significantly lower number of hospitalizations and nursing home visits among telemonitored patients (*p *< 0.05) and generated savings of $343 per telemonitored patient ($2378) compared with the control group ($2688) [19]. Similarly, a cost analysis using a telecare system for patients with diabetes showed savings of $1065 per patient per year (PPPY) [20]. A large part of the savings attributable to these types of programs are from reductions in hospitalizations.

Though often difficult to measure in monetary terms, an important value dimension of PMT-supported independent living is health-related quality of life (HRQoL). For example, Karakaya et al. examined functioning and HRQoL differences between independent living and nursing homes, finding that nursing home residents generally exhibited better functioning but had more depressive symptoms and lower overall HRQoL [22]. Similar results were reported by Kostka et al., who found that HRQoL decreased with a growing level of dependence and institutionalization [23]. Other studies, using a variety of methods, have reached very consistent results [24–26]. Within community-dwelling elderly, the literature very consistently shows higher levels of HRQoL among those using some form of PMT compared to those not using such technologies [27–33].

## Methods

### Conceptual framework

In this next section, we describe the economic value framework used to identify the main cost savings pathways and cost variables associated with independent living supported by PMT versus two alternatives: independent living supported by the “standard of care” (“SOC”) and facility-based care. We further assume a target population of “long-term care eligible” individuals with moderate or intermediate levels of comorbidities. The reason for these latter inclusion criteria is that individuals with above-average comorbidities would likely require monitoring and care that would transcend PMT (i.e., obscuring the “PMT effect” from other disease management and treatment effects).

**Fig. 1:**
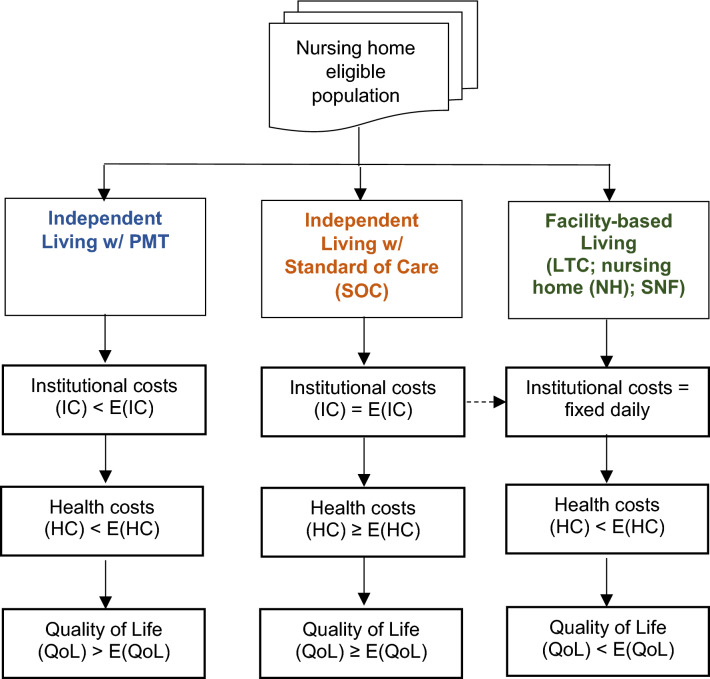
PMT value framework

For each of these three pathways, we examine the impact of each pathway in three dimensions: (1) institutional costs (i.e., the costs associated with requiring institutional long-term care, such as in skilled nursing facilities (SNFs) and other types of long-term care facilities (i.e., independent living versus non-independent living); (2) health care costs (i.e., outpatient visits, including emergency room, and inpatient hospitalizations); and (3) HRQoL. The relationships and expected outcomes of each of these value elements are shown in Fig. 1 for each of the three management pathways.

As discussed in the background section, monitoring systems have the potential to allow seniors to use institutional care (e.g., short-term facility-based nursing services, such as skilled nursing facilities, as well as longer-term services, such as nursing homes) more parsimoniously and this in turn is likely to generate direct savings. Such monitoring has been shown to result in saved or avoided “facility days,” the value of which can be linked to the expected daily, monthly, or annual costs of those services.

The expected costs of intervention “x” may be expressed as *E* (*C*_x_) and are the product of the probability that “x” occurs (*p*_x_) and the average or marginal costs of “x” (denoted as C_x_); that is, *E* (*C*_x_) = *p*_x_ * *C*_x_. Referring to Fig. 1, the hypothesis is that any intervention intended to support independent living (such as PMT) has the potential to reduce the probability of use of institutional care providers, such that IC_LHM_ < *E* (IC). In the case of the SOC-independent living, we hypothesize that individuals face an average or normal probability of institutional usage, such that IC_SOC_ = *E* (IC). For the facility pathway, *p*_x_ = 1.0, thus *E* (IC) is equal to the direct costs of institutional care. Note that the dashed hour connecting the SOC pathway and the institutional pathway suggests that “poorly executed” independent living support (which may be the case with some forms of SOC) can increase the probability of transitioning from the SOC arm to the facility arm (thereby increasing the total expected costs of the SOC arm).

The second way in which monitoring systems such as PMT generate value is by potentially reducing the probability of incurring other kinds of health care costs. Monitoring and signaling have the potential to initiate health-related interventions (e.g., from an informal caregiver, a case manager, or a clinician) at less costly stages, thereby helping to reduce unnecessary use of more resource-intensive settings, such as emergency rooms (ERs) and inpatient hospitalizations. A related value comes from LHM potentially shortening response time for “adverse events.” For some adverse events, such as stroke and heart attack, emergency response time (i.e., the time from call to arrival at ER) is highly associated with outcomes, including mortality.

Referring back to the health care cost component of Fig. 1, the hypothesis is that any intervention intended to support independent living (such as PMT) has the potential to reduce the probability of use of other kinds of care resources, such as ER visits and hospitalizations. That is, for health care costs (HC) on the PMT pathway, HC_LHM_ < *E* (HC). In the case of the SOC-independent living, we hypothesize that individuals face an average or normal probability of health care usage, such that HC_SOC_ ≥ *E* (HC). On the SOC pathway, the expected costs may in some cases be higher than normal because of the limitations of existing SOC approaches in supporting independent living, and the fact that avoidable exacerbating events lead to not only a hospitalization, but in some cases can also lead to recovery in an SNF or longer term facility. For the facility pathway, *E* (HC) is expected to be lower than normal because (at least in theory and according to some literature) the fill-time intensive monitoring associated with institutional settings lowers the use of more intensive community-based providers.^1^

The third way in which monitoring systems can add value is by enhancing the HRQoL of individuals. Consistent with the literature, we hypothesize that the independent living options, as opposed to institutional care, are associated with higher expected HRQoL. Specifically, we hypothesize that HRQoL will be higher than normal in the PMT group, greater than or equal to normal in the SOC group, and lowest in the institutional setting group. In some cases, HRQoL can be “mapped” to costs and incorporated directly into an economic model. Another role for HRQoL can be to generate “utilities” to weight (or “quality adjust”) survival, as measured by life-years. In our model, however, we include it only as a descriptive adjunct; we do not convert HRQoL data into dollar terms.

### Cost–benefit model

We constructed a cost–benefit analysis (CBA) of PMT based on the conceptual model put forth above in Fig. 1. For each of the three intervention options (or “arms”), we summed the institutional costs and the health care costs associated with each arm. All outcomes, including costs and benefits, were measured in dollar terms. Model data (described in the next section) consisted of probabilities, rates, and costs drawn from the literature (some of which is cited above) and GreatCall, the makers of LHM. The CBA has several important supporting assumptions: (1) patients on the two independent living arms (PMT and SOC) have a non-zero probability of transitioning between independent living and institutional care (mainly SNF), and this transition can be modeled using a Markov process; and (2) the set of health care costs considered in the model will be limited by the areas in which PMT is expected to have an impact via its monitoring capacity. Examples of the latter, in the case of LHM, are shown in Table 1.

**Table 1:** LHM tracking sensor activity capabilities and list of identifiable conditions

Tracking sensor activities for ADLs	Relevant and related diseases, conditions, and symptoms
Sleep	Acute bronchitis and bronchiolitis, COPD, CHF, Arthritis, Pain, Medications, UTI, Depression/Anxiety, Vomiting, Diarrhea, Edema
Location	COPD, Diabetes mellitus, Heart Disease, Depression/Anxiety
Bathroom use	Diabetes mellites, UTI, Edema, CHF, IBS, Diarrhea, Constipation, Crohn’s, Dehydration
Gait	Arthritis, Asthma, Chronic bronchitis, CHF, COPD, Pain, Edema
Movement	Acute bronchitis and bronchiolitis, Arthritis, Asthma, Chronic bronchitis, COPD, Heart disease, Depression/Anxiety, Pneumonia, Pain, Edema, Fatigue
Social	Asthma, Chronic bronchitis, Heart Disease, Depression/Anxiety, Pain, SOB, Fatigue
Eating	Acute bronchitis and bronchiolitis, COPD, Diabetes mellitus, Major depressive disorder, Pneumonia, Nausea, Constipation, Heart Disease
Vitals	Acute bronchitis and bronchiolitis, Asthma, Chronic bronchitis, CHF, COPD, Chronic renal failure, Diabetes mellitus, Heart Disease, Pneumonia, Infection

*Source* GreatCall data on LHM product

Using data from the National Hospital Discharge Survey, CDC/NCHS National Hospital Ambulatory Medical Care Survey, CDC National Health Data, and Medicare Utilization and Payment data, we extracted data on the national population and those ages 65 years and over, including data on probability of use of medical care in inpatient and outpatient settings for each disease variable listed in Table 1, and the prevalence of disease in the U.S. population (see Appendix A in Online Resource 1). Average cost for annual utilization use was generated using data from the literature, cited above.

The probability of an inpatient or outpatient event associated with each disease variable was calculated by multiplying the probability of health care resource use and the prevalence of the disease in the U.S. population, stratified by age and total US population to create a range of values for high and low resource utilization in the entire insured population. The estimated cost of care for the entire cohort for inpatient admissions was then added to the total estimated cost of SNF days after an inpatient admission (for the specific diseases); according to the literature, the mean length of stay is 3 days for the entire hospitalized population (Table 2).

**Table 2:** Cost data for cost–benefit analysis by setting and length of stay

Variables	Cost per use
Institutional costs	
SNF (per day)^a^	$292
Nursing home (per day)^a^	$267
Health care costs	
Ambulatory outpatient visit (per visit)^b^	$277
Hospital Outpatient services (per visit)^b^	$1777
Outpatient Emergency Services (per visit)^b^	$1285
Inpatient admission (per day)^c^	$1857–$2372

^a^Costs obtained from www.genworth.com

^b^Cost obtained from U.S. AHRQ

^c^Cost obtained from Becker’s Hospital Review average costs per inpatient day, by state

We then added estimated annual nursing home utilization costs (assuming 5.5% of SNF patients will go to nursing home) for the hospitalized population to generate an annual total cost of the standard of care for the insured population (also see Appendix B and Appendix C in Online Resources 2 and 3). According to the literature, about 30% of the population has above average or high pharmacy resource utilization. We applied this cost to the overall population, taking into account some proportion will be healthy individuals. We generated ER service utilization combining CDC data on ER utilization and ambulance transport statistics from hospital systems. This generated the total cost of ER services and ambulance services in the national population. In addition, we assumed that 10% of the overall population would have utilized nursing homes because of their monitoring needs, but who otherwise would have been served by PMT. It is important to note that all costs are estimates and may vary depending on disease burden and payer population; these estimates are generated using data from the national population (i.e., all ages) and the aged 65 and over population to create a range of data appropriate to potential PMT users.

Using cost savings estimates from the Finch et al. study of members of the Fallon NaviCare Plan (Massachusetts) [34], we generated estimates of cost of care for an identical cohort of potential PMT subscribers by applying the estimated percent changes to respective service lines such as inpatient admissions, outpatient visits, emergency department use including ambulance services, long-term care use, skilled nursing facility use, and pharmacy utilization. The PMT arm was calculated using service line reduction percentages (Table 3) for each respective service arm. We then totaled inpatient, outpatient, pharmacy, emergency services, and nursing home utilization to create two arms: SOC vs. PMT.

**Table 3:** Expected change in costs attributable to PMT

Service line	Percent change (%)
Inpatient	− 22
Emergency department	− 31
Long-term care	− 62
Skilled nursing facility	− 38
Ambulance	− 12
Home care	+ 7
Outpatient	+ 5
Pharmacy	+ 2
Other	− 9
Total	− 15

Service line reductions obtained from Finch study [34]. The Finch et al. study was a cost analysis of assisted living technology with 268 dual-eligible Medicare and Medicaid enrollees. For the purposes of this analysis we averaged the percent change for the group that declined GreatCall services and the historical comparison group

These calculations explained above created a high and low range of costs for the aged 65 and over population and national populations for individuals in the SOC and PMT arms. The annual cost of a PMT subscription (assumed to be $960) was then added to the cost of care for those on the PMT arm of the model. Cost savings for the great call arm were generated by subtracting the total cost of the standard of care for the population and the total cost of the great call arm for each of the populations. Per-member per-year (PMPY) and per-member per-month (PMPM) savings from PMT were then generated using this range of data.

## Results

The cost–benefit model showed savings between $418 and $425 (PMPM) in comparison to members who are on the current “standard-of-care” pathway. We performed the same extrapolation to 1000 patients which resulted in savings for an insurance provider up to $5100 PMPY and $5,100,277 for the entire 1000-person cohort annually. Table 4 depicts an average estimated annual savings for a population of 1000–5000 individuals who are on the PMT service, compared to those who are not.

**Table 4:** Projected savings for 10% of PMT arm tracking with the standard of care arm

Population size	Estimated annual savings 100% success^a^	Estimated annual savings 90% Success^b^
1000	$5.1 million	$4.5 million
2000	$10.2 million	$9.1 million
3000	$15.3 million	$13.7 million
4000	$20.4 million	$18.3 million
5000	$25.5 million	$22.9 million

^a^Savings if all individuals with the population successfully track according to the assumptions made in the model

^b^Estimated savings if 10% of individuals within each population size fail to track with the PMT arm and subscribers re-enter the standard of care arm

Sensitivity analysis of the model demonstrated that the model was most sensitive to the change in preventable nursing home utilization defined as residents who are in nursing home care due to lack of home care resources for monitoring (i.e., that could otherwise be addressed by PMT). For example, 5% increase in preventable nursing home utilization generates a PMPM cost savings of over 30% for the target population.

## Discussion

The cost–benefit model results were expressed as the total “cost savings” (i.e., probability multiplied by costs for each component) of a healthcare-associated event for each intervention arm: PMT, the current standard of care, or institutional care. Total expected cost savings do not necessarily depict an actual cost to a provider; instead, they represent the expected cost for a typical population given the population characteristics. The total projected cost savings for individuals that are on the PMT arm are projected to be upwards of $425 PMPM, with annual savings of $5069 PMPY, and over $5.1 million for a target population of 1000 individuals who successfully track through the PMT service.

These results have also been demonstrated in practice. For example, in 2016, PMT maker GreatCall partnered with Centene Corporation’s Bridgeway Long-Term Care (LTC) health plan in Arizona to offer passive monitoring technology to seniors with multiple comorbidities living independently in their own homes. GreatCall provides remote monitoring systems (e.g., motion, contact, bed, and toilet sensors) and analytic services to alert caregivers when a member experiences changes in patterns of daily living. Upon receiving an alert, GreatCall and health plan care managers work together to intervene before this event results in an avoidable, costly transition in care. Study results were positive and suggest that the GreatCall passive monitoring and alert system holds promise in reducing utilization and spending among seniors living independently. However, given the small sample size, the small proportion of randomized members who were successfully recruited for installation, and the high attrition rate, these results must be interpreted with caution. Further research with a larger population and a more rigorous randomization process is recommended.

In recent years, there has been a shift in US markets to a population-based perspective when assessing the costs and benefits of a healthcare intervention. Cost–benefit analysis results can be variable when looking at the perspective of a health system or accountable care organization. In the current model structure, population characterizes may heavily impact cost savings—and greater savings may be seen in PMT subscribers who have higher rates of healthcare utilization.

A related question is whether these results could be generally applicable to countries outside of the US, where health systems typically adopt a population-based (i.e., “societal”) perspective on economic evaluation. Such systems typically consider factors such as informal care (for example, the costs incurred by family members in caring for the individual, including a proxy of caregiver’s hourly wage), which in this framework we have left out. Were informal care to be considered, it is likely that the cost savings associated with PMT would be higher than reported here. It is also possible that other societal factors could be considered, such as the indirect benefits associated with lower rates of depression among the elderly.

## Conclusions

We employed a simple cost–benefit simulation model to estimate the likely cost impact of individuals enrolling in PMT services. The expected cost calculations clearly demonstrate the value of PMT and show the potential value to payers and integrated delivering systems in offering to PMT to individuals who are likely to benefit the most from the services.

## Electronic supplementary material

Below is the link to the electronic supplementary material.

Supplementary material 1 (DOCX 14 kb)

Supplementary material 2 (DOCX 25 kb)

Supplementary material 3 (DOCX 24 kb)

## Data Availability

All data generated or analyzed during this study are included in this published article.

## Footnotes

1 For example, a patient who may be experiencing severe edema increases their intake of fluid pills to relieve swelling in his legs. The fluid pills cause an increase in bathroom utilization, but do not directly treat or address the edema. If the patient is using LHM, changes in ADL patterns may indicate the need for intervention, and the individual can thus be treated before the edema becomes an infection. In contrast, an SOC patient may experience progression of symptoms leading to inpatient treatment for edema progressed to an infection and may need to be admitted into an SNF or nursing home to complete the treatment.

## References

[1] Murray CJ, Abraham J, Ali MK (2013). The state of US health, 1990–2010: burden of diseases, injuries, and risk factors. JAMA.

[2] WHO (2011) Global health and aging. http://www.who.int/aging/publications/global_health.pdf. Accessed 14 Nov 2017

[3] UN (2013) Aging. http://www.un.org/en/globalissues/aging/. Accessed 13 Nov 2017

[4] Liu L, Stroulia E, Nikolaidis I (2016). Smart homes and home health monitoring technologies for older adults: a systematic review. Int J Med Inform.

[5] Czaja SJ (2017). The potential role of technology in supporting older adults. Public Policy Aging Rep.

[6] Smith A, Bensink M, Armfield N (2005). Telemedicine and rural health care applications. J Postgrad Med.

[7] Bashshur RL, Shannon GW, Krupinski EA (2009). National telemedicine initiatives: essential to healthcare reform. Telemed J e-Health.

[8] Rosenthal TC, Williams ME, Naughton BJ (2006). Office care geriatrics.

[9] Marengoni A, Angleman S, Melis R (2011). Aging with multimorbidity: a systematic review of the literature. Ageing Res Rev.

[10] Rechel B, Grundy E, Robine JM (2013). Ageing in the European union. Lancet (Lond, Engl).

[11] Gale CR, Baylis D, Cooper C (2013). Inflammatory markers and incident frailty in men and women: the English Longitudinal Study of Ageing. Age (Dordr, Neth).

[12] Cherniack EP, Florez HJ, Troen BR (2007). Emerging therapies to treat frailty syndrome in the elderly. Altern Med Rev.

[13] Kearns WD, Fozard JL, Becker M (2012). Path tortuosity in everyday movements of elderly persons increases fall prediction beyond knowledge of fall history, medication use, and standardized gait and balance assessments. J Am Med Dir Assoc.

[14] Merriel SW, Andrews V, Salisbury C (2014). Telehealth interventions for primary prevention of cardiovascular disease: a systematic review and meta-analysis. Prev Med.

[15] Redfern J, Usherwood T, Harris MF (2014). A randomised controlled trial of a consumer-focused e-health strategy for cardiovascular risk management in primary care: the consumer navigation of electronic cardiovascular tools (CONNECT) study protocol. BMJ Open.

[16] Cruz J, Brooks D, Marques A (2014). Home telemonitoring effectiveness in COPD: a systematic review. Int J Clin Pract.

[17] Purcell R, McInnes S, Halcomb EJ (2014). Telemonitoring can assist in managing cardiovascular disease in primary care: a systematic review of systematic reviews. BMC Fam Pract.

[18] Finkelstein SM, Speedie SM, Potthoff S (2006). Home telehealth improves clinical outcomes at lower cost for home healthcare. Telemed J e-Health.

[19] Pare G, Sicotte C, St-Jules D (2006). Cost-minimization analysis of a telehomecare program for patients with chronic obstructive pulmonary disease. Telemed J e-Health.

[20] Biermann E, Dietrich W, Rihl J (2002). Are there time and cost savings by using telemanagement for patients on intensified insulin therapy? A randomised, controlled trial. Comput Methods Progr Biomed.

[21] Callan A, O’Shea E (2015). Willingness to pay for telecare programmes to support independent living: results from a contingent valuation study. Soc Sci Med.

[22] Karakaya MG, Bilgin SC, Ekici G (2009). Functional mobility, depressive symptoms, level of independence, and quality of life of the elderly living at home and in the nursing home. J Am Med Dir Assoc.

[23] Kostka T, Jachimowicz V (2010). Relationship of quality of life to dispositional optimism, health locus of control and self-efficacy in older subjects living in different environments. Qual Life Res Int J Qual Life Asp Treat Care Rehabil.

[24] Chin L, Quine S (2012). Common factors that enhance the quality of life for women living in their own homes or in aged care facilities. J Women Aging.

[25] Nikmat AW, Hawthorne G, Al-Mashoor SH (2015). The comparison of quality of life among people with mild dementia in nursing home and home care–a preliminary report. Dementia (Lond, Engl).

[26] Olsen C, Pedersen I, Bergland A (2016). Differences in quality of life in home-dwelling persons and nursing home residents with dementia—a cross-sectional study. BMC Geriatr.

[27] Graybill EM, McMeekin P, Wildman J (2014). Can aging in place be cost effective? A systematic review. PLoS One.

[28] Gustafson DH, McTavish F, Gustafson DH (2015). The effect of an information and communication technology (ICT) on older adults’ quality of life: study protocol for a randomized control trial. Trials.

[29] Hirani SP, Beynon M, Cartwright M (2014). The effect of telecare on the quality of life and psychological well-being of elderly recipients of social care over a 12-month period: the whole systems demonstrator cluster randomised trial. Age Ageing.

[30] Matlabi H, Parker SG, McKee K (2011). The contribution of home-based technology to older people’s quality of life in extra care housing. BMC Geriatr.

[31] Muncert ES, Bickford SA, Guzic BL (2011). Enhancing the quality of life and preserving independence for target needs populations through integration of assistive technology devices. Telemed J e-Health.

[32] Patil R, Uusi-Rasi K, Kannus P (2014). Concern about falling in older women with a history of falls: associations with health, functional ability, physical activity and quality of life. Gerontology.

[33] Zhang Q, Karunanithi M, Rana R, et al (2013) Determination of activities of daily living of independent living older people using environmentally placed sensors. Conference proceedings: Annual International Conference of the IEEE Engineering in Medicine and Biology Society IEEE Engineering in Medicine and Biology Society Annual Conference 2013:7044–7047. 10.1109/embc.2013.6611180 10.1109/EMBC.2013.6611180 24111367

[34] Finch M, Griffin K, Pacala JT (2017). Reduced healthcare use and apparent savings with passive home monitoring technology: a pilot study. J Am Geriatr Soc.

