# Supplementary material for: Economic evaluation of passive monitoring technology for seniors
Source: Aging Clin Exp Res. 2019 Sep 14;32(7):1375–82. doi: 10.1007/s40520-019-01323-2 (PMC7316690; doi:10.1007/s40520-019-01323-2)
Supplement: Supplementary file 1 — Supplementary material 1 (DOCX 14 kb) [file 40520_2019_1323_MOESM1_ESM.docx]

**APPENDIX A**

Population Specific Probabilities for Inpatient and Outpatient Hospital Admissions and Disease Prevalence in the United States

| **Cost Data and Probabilities for Inpatient and Outpatient Settings, By Common Condition** | | | | | | | |
| --- | --- | --- | --- | --- | --- | --- | --- |
| **Common Condition** | **National Inpatient Probability (2014) (a)** | **65+ Inpatient Probability (2010) (a)** | **National Outpatient Probability (2011) (b)** | **65+ Outpatient Probability (2011) (b)** | **Prevalence in US (low) (c)** | **Prevalence in US (high) (c)** | **Average Inpatient Cost per Episode (d)** |
| Acute bronchitis^1^ | 0.000916 | 0.000596 | 0.080050 | 0.090050 | 0.000500 | 0.045993 | $5,317 |
| Arthritis^4^ | 0.003390 | 0.014720 | 0.000084 | 0.062000 | 0.003180 | 0.049879 | $5,398 |
| Asthma^1^ | 0.006176 | 0.002558 | 0.000043 | 0.023000 | 0.000804 | 0.076923 | $3,790 |
| Chronic bronchitis^1^ | 0.000010 | 0.009884 | 0.000031 | 0.000041 | 0.001500 | 0.030122 | $4,512 |
| Chronic heart Failure^2^ | 0.013186 | 0.017880 | 0.000012 | 0.011000 | 0.002100 | 0.018462 | $6,143 |
| COPD^1^ | 0.000010 | 0.009884 | 0.000031 | 0.026000 | 0.000500 | 0.045993 | $7,061 |
| Chronic renal failure^6^ | 0.000003 | 0.000004 | 0.000014 | 0.017000 | 0.002030 | 0.097167 | $5,786 |
| Diabetes mellitus^3^ | 0.017722 | 0.004917 | 0.000007 | 0.080000 | 0.004300 | 0.323891 | $5,236 |
| Hypertension^2^ | 0.037519 | 0.002508 | 0.016185 | 0.168000 | 0.005640 | 0.242918 | $6,266 |
| Ischemic heart disease^2^ | 0.002141 | 0.001142 | 0.000108 | 0.021000 | 0.002970 | 0.091985 | $11,550 |
| Mental Health disorder^5^ | 0.002670 | 0.003670 | 0.021460 | 0.039000 | 0.001300 | 0.076000 | $4,259 |
| Pneumonia^1^ | 0.006622 | 0.015422 | 0.000049 | 0.000060 | 0.043412 | 0.002915 | $8,698 |
| Urinary tract infection^6^ | 0.008808 | 0.010430 | 0.046571 | 0.056571 | 0.025911 | 0.133200 | $4,700 |
| Digestive System | 0.008451 | 0.009845 | 0.018964 | 0.006950 | 0.000400 | 0.210529 | $8,200 |
| Skin | 0.001273 | 0.002127 | 0.011812 | 0.000870 | 0.000800 | 0.275308 | $8,365 |
| Fatigue | 0.000035 | 0.000045 | 0.012373 | 0.022373 | 0.000600 | 0.000148 | $4,800 |
| *Notes:* a. Data utilized to calculate probability was obtained from National Hospital Discharge Survey (2010) [66]; b. Data obtained to calculate probability was obtained from CDC/NCHS, National Hospital Ambulatory Medical Care Survey [67]; c: Statistics obtained from CDC d. Values obtained from CMS 2015 Medicare Utilization and Payment Data [65] 1. Diseases of the respiratory system; 2. Cardiovascular disease; 3. Endocrine, nutritional and metabolic diseases and immunity disorders; 4. Musculoskeletal Disorders; 5. Mental Health Disorder; 6. Excretory System; In cases where data was not available for 65+ population, national averages are used. | | | | | | | |
