# Supplementary material for: Economic evaluation of passive monitoring technology for seniors
Source: Aging Clin Exp Res. 2019 Sep 14;32(7):1375–82. doi: 10.1007/s40520-019-01323-2 (PMC7316690; doi:10.1007/s40520-019-01323-2)
Supplement: Supplementary file 3 — Supplementary material 3 (DOCX 24 kb) [file 40520_2019_1323_MOESM3_ESM.docx]

**APPENDIX C**

Tables of Cost Estimates for Cohort Size of 1,000 in the PMT Arm

| **Inpatient Cost Estimates for Overall Cohort Using Low Disease Prevalence (65+ population)** | | | | |
| --- | --- | --- | --- | --- |
| **Disease** | **PMT Hospital Cost for Cohort** | **PMT SNF Cost** | **PMT Nursing Home Cost** | **Estimated Cost w/PMT** |
| Acute bronchitis^1^ | $1.24 | $0.16 | $0.61 | $2.02 |
| Arthritis^4^ | $197.98 | $25.35 | $96.46 | $319.79 |
| Asthma^1^ | $6.11 | $1.11 | $4.24 | $11.46 |
| Chronic bronchitis^1^ | $52.41 | $8.03 | $30.55 | $90.99 |
| Chronic heart Failure^2^ | $180.73 | $20.33 | $77.38 | $278.44 |
| COPD^1^ | $27.34 | $2.68 | $185.16 | $215.18 |
| Chronic renal failure^6^ | $0.04 | $0.00 | $0.02 | $0.06 |
| Diabetes mellitus^3^ | $86.74 | $11.45 | $43.57 | $141.76 |
| Hypertension^2^ | $69.45 | $7.66 | $29.15 | $106.26 |
| Ischemic heart disease^2^ | $30.70 | $1.84 | $6.99 | $39.53 |
| Mental Health disorder^5^ | $15.92 | $2.58 | $9.83 | $28.34 |
| Pneumonia^1^ | $4562.63 | $362.57 | $1379.65 | $6304.85 |
| Urinary tract infection^6^ | $995.24 | $146.36 | $556.93 | $1698.53 |
| Digestive System | $25.30 | $2.13 | $8.12 | $35.55 |
| Skin | $11.15 | $0.92 | $3.51 | $15.58 |
| Fatigue | $0.10 | $0.01 | $0.06 | $0.17 |
| Note: All costs in tis table are estimates and may vary depending on disease burden in specific payor population. These estimates are generated using disease burden and healthcare utilization data from the National and 65-year old + population in the United States, to create a range of data for the nursing home eligible population in the United States. Reference data can be found in Appendix A. | | | | |

| **Inpatient Cost Estimates for Overall Cohort Using High Disease Prevalence (65+ Population)** | | | | |
| --- | --- | --- | --- | --- |
| **Disease** | **PMT Hosp Cost for Cohort** | **PMT SNF Cost** | **PMT Nursing Home Cost** | **Estimated Cost w/PMT** |
| Acute bronchitis^1^ | $114.20 | $14.85 | $56.49 | $185.53 |
| Arthritis^4^ | $3105.36 | $397.62 | $1513.04 | $5016.02 |
| Asthma^1^ | $584.28 | $106.56 | $405.47 | $1096.31 |
| Chronic bronchitis^1^ | $1052.51 | $161.23 | $613.52 | $1827.26 |
| Chronic heart Failure^2^ | $1588.83 | $178.77 | $680.25 | $2447.85 |
| COPD^1^ | $2.51 | $0.25 | $0.94 | $3.70 |
| Chronic renal failure^6^ | $1.76 | $0.21 | $0.80 | $2.77 |
| Diabetes mellitus^3^ | $6533.64 | $862.48 | $3281.91 | $10678.03 |
| Hypertension^2^ | $2991.32 | $329.96 | $1255.58 | $4576.87 |
| Ischemic heart disease^2^ | $950.93 | $56.91 | $216.54 | $1224.38 |
| Mental Health disorder^5^ | $930.76 | $151.05 | $574.78 | $1656.59 |
| Pneumonia^1^ | $306.37 | $24.35 | $92.64 | $423.35 |
| Urinary tract infection^6^ | $5116.14 | $752.38 | $2862.97 | $8731.49 |
| Digestive System | $13316.74 | $1122.48 | $4271.26 | $18710.48 |
| Skin | $3838.54 | $317.17 | $1206.90 | $5362.61 |
| Fatigue | $0.02 | $0.00 | $0.01 | $0.04 |
| Note: All costs in tis table are estimates and may vary depending on disease burden in specific payor population. These estimates are generated using disease burden and healthcare utilization data from the National and 65-year old + population in the United States, to create a range of data for the nursing home eligible population in the United States. Reference data can be found in Appendix A. | | | | |

| **Inpatient Cost Estimates for Overall Cohort Using Low Disease Prevalence (National Population)** | | | | |
| --- | --- | --- | --- | --- |
| **Disease** | **PMT Hosp Cost for Cohort** | **PMT SNF Cost** | **PMT Nursing Home Cost** | **Estimated Cost w/PMT** |
| Acute bronchitis^1^ | $1.91 | $0.25 | $0.94 | $3.10 |
| Arthritis^4^ | $45.59 | $5.84 | $22.22 | $73.65 |
| Asthma^1^ | $14.74 | $2.69 | $10.23 | $27.67 |
| Chronic bronchitis^1^ | $0.05 | $0.01 | $0.03 | $0.09 |
| Chronic heart Failure^2^ | $133.28 | $15.00 | $57.06 | $205.33 |
| COPD^1^ | $0.03 | $0.00 | $0.01 | $0.04 |
| Chronic renal failure^6^ | $0.02 | $0.00 | $0.01 | $0.04 |
| Diabetes mellitus^3^ | $312.63 | $41.27 | $157.04 | $510.93 |
| Hypertension^2^ | $1038.88 | $114.60 | $436.06 | $,589.54 |
| Ischemic heart disease^2^ | $57.56 | $3.44 | $13.11 | $74.11 |
| Mental Health disorder^5^ | $11.58 | $1.88 | $7.15 | $20.62 |
| Pneumonia^1^ | $1959.06 | $155.68 | $592.38 | $2707.11 |
| Urinary tract infection^6^ | $840.47 | $123.60 | $470.32 | $1434.39 |
| Digestive System | $21.72 | $1.83 | $6.97 | $30.52 |
| Skin | $6.68 | $0.55 | $2.10 | $9.33 |
| Fatigue | $0.08 | $0.01 | $0.04 | $0.13 |
| Note: All costs in tis table are estimates and may vary depending on disease burden in specific payor population. These estimates are generated using disease burden and healthcare utilization data from the National and 65-year old + population in the United States, to create a range of data for the nursing home eligible population in the United States. Reference data can be found in Appendix A. | | | | |

| **Inpatient Cost Estimates for Overall Cohort Using High Disease Prevalence (National Population)** | | | | |
| --- | --- | --- | --- | --- |
| **Disease** | **PMT Hosp Cost for Cohort** | **PMT SNF Cost** | **PMT Nursing Home Cost** | **Estimated Cost w/PMT** |
| Acute bronchitis^1^ | $175.55 | $22.82 | $86.84 | $285.20 |
| Arthritis^4^ | $715.16 | $91.57 | $348.45 | $1155.18 |
| Asthma^1^ | $1410.69 | $257.27 | $978.96 | $2646.92 |
| Chronic bronchitis^1^ | $1.06 | $0.16 | $0.62 | $1.84 |
| Chronic heart Failure^2^ | $1171.68 | $131.83 | $501.65 | $1805.17 |
| COPD^1^ | $0.00 | $0.00 | $0.00 | $0.00 |
| Chronic renal failure^6^ | $1.13 | $0.14 | $0.51 | $1.78 |
| Diabetes mellitus^3^ | $23548.09 | $3108.50 | $11828.45 | $38485.04 |
| Hypertension^2^ | $44745.45 | $4935.74 | $18781.50 | $68462.69 |
| Ischemic heart disease^2^ | $1782.57 | $106.67 | $405.92 | $2295.16 |
| Mental Health disorder^5^ | $677.15 | $109.89 | $418.16 | $1205.21 |
| Pneumonia^1^ | $131.55 | $10.45 | $39.78 | $181.78 |
| Urinary tract infection^6^ | $4320.52 | $635.38 | $2417.74 | $7373.64 |
| Digestive System | $11431.15 | $963.54 | $3666.47 | $16061.16 |
| Skin | $2297.17 | $189.81 | $722.27 | $3209.25 |
| Fatigue | $0.02 | $0.00 | $0.01 | $0.03 |
| Note: All costs in tis table are estimates and may vary depending on disease burden in specific payor population. These estimates are generated using disease burden and healthcare utilization data from the National and 65-year old + population in the United States, to create a range of data for the nursing home eligible population in the United States. Reference data can be found in Appendix A. | | | | |

| **Outpatient Cost Estimates for Overall Cohort Using Low Disease Prevalence (65+ Population)** | |
| --- | --- |
| **Disease** | **PMT Hospital Cost for Cohort** |
| Acute bronchitis^1^ | $84.13 |
| Arthritis^4^ | $368.38 |
| Asthma^1^ | $34.55 |
| Chronic bronchitis^1^ | $0.11 |
| Chronic heart Failure^2^ | $43.16 |
| COPD^1^ | $24.29 |
| Chronic renal failure^6^ | $64.48 |
| Diabetes mellitus^3^ | $642.74 |
| Hypertension^2^ | $1770.38 |
| Ischemic heart disease^2^ | $116.53 |
| Mental Health disorder^5^ | $94.73 |
| Pneumonia^1^ | $4.87 |
| Urinary tract infection^6^ | $2738.82 |
| Digestive System | $5.19 |
| Skin | $1.30 |
| Fatigue | $25.08 |
| Note: All costs in tis table are estimates and may vary depending on disease burden in specific payor population. These estimates are generated using disease burden and healthcare utilization data from the National and 65-year old + population in the United States, to create a range of data for the nursing home eligible population in the United States. Reference data can be found in Appendix A. | |

| **Outpatient Cost Estimates for Overall Cohort Using High Disease Prevalence (65+ Population)** | |
| --- | --- |
| **Disease** | **PMT Hosp Cost for Cohort** |
| Acute bronchitis^1^ | $7738.36 |
| Arthritis^4^ | $5778.16 |
| Asthma^1^ | $3305.69 |
| Chronic bronchitis^1^ | $2.30 |
| Chronic heart Failure^2^ | $379.44 |
| COPD^1^ | $2234.29 |
| Chronic renal failure^6^ | $3086.36 |
| Diabetes mellitus^3^ | $48413.56 |
| Hypertension^2^ | $76251.36 |
| Ischemic heart disease^2^ | $3609.23 |
| Mental Health disorder^5^ | $5538.04 |
| Pneumonia^1^ | $0.33 |
| Urinary tract infection^6^ | $14079.22 |
| Digestive System | $2733.85 |
| Skin | $447.52 |
| Fatigue | $6.19 |
| Note: All costs in tis table are estimates and may vary depending on disease burden in specific payor population. These estimates are generated using disease burden and healthcare utilization data from the National and 65-year old + population in the United States, to create a range of data for the nursing home eligible population in the United States. Reference data can be found in Appendix A. | |

| **Outpatient Cost Estimates for Overall Cohort Using Low Disease Prevalence (National Population)** | |
| --- | --- |
| **Disease** | **PMT Hosp Cost for Cohort** |
| Acute bronchitis^1^ | $74.78 |
| Arthritis^4^ | $0.50 |
| Asthma^1^ | $0.06 |
| Chronic bronchitis^1^ | $0.09 |
| Chronic heart Failure^2^ | $0.05 |
| COPD^1^ | $0.03 |
| Chronic renal failure^6^ | $0.05 |
| Diabetes mellitus^3^ | $0.06 |
| Hypertension^2^ | $170.56 |
| Ischemic heart disease^2^ | $0.60 |
| Mental Health disorder^5^ | $52.13 |
| Pneumonia^1^ | $3.96 |
| Urinary tract infection^6^ | $2254.69 |
| Digestive System | $14.17 |
| Skin | $17.66 |
| Fatigue | $13.87 |
| Note: All costs in tis table are estimates and may vary depending on disease burden in specific payor population. These estimates are generated using disease burden and healthcare utilization data from the National and 65-year old + population in the United States, to create a range of data for the nursing home eligible population in the United States. Reference data can be found in Appendix A. | |

| **Outpatient Cost Estimates for Overall Cohort Using High Disease Prevalence (National Population)** | |
| --- | --- |
| **Disease** | **PMT Hosp Cost for Cohort** |
| Acute bronchitis^1^ | $6879.02 |
| Arthritis^4^ | $7.86 |
| Asthma^1^ | $6.20 |
| Chronic bronchitis^1^ | $1.74 |
| Chronic heart Failure^2^ | $0.41 |
| COPD^1^ | $2.66 |
| Chronic renal failure^6^ | $2.51 |
| Diabetes mellitus^3^ | $4.44 |
| Hypertension^2^ | $7345.93 |
| Ischemic heart disease^2^ | $18.48 |
| Mental Health disorder^5^ | $3047.37 |
| Pneumonia^1^ | $0.27 |
| Urinary tract infection^6^ | $11590.47 |
| Digestive System | $7459.62 |
| Skin | $6076.19 |
| Fatigue | $3.42 |
| Note: All costs in tis table are estimates and may vary depending on disease burden in specific payor population. These estimates are generated using disease burden and healthcare utilization data from the National and 65-year old + population in the United States, to create a range of data for the nursing home eligible population in the United States. Reference data can be found in Appendix A. | |
